# Necrotizing Fasciitis of the Thigh as Unusual Colonoscopic Polypectomy Complication: Review of the Literature with Case Presentation

**DOI:** 10.3390/medicina58010131

**Published:** 2022-01-15

**Authors:** Sara Sablone, Elpiniki Lagouvardou, Gerardo Cazzato, Francesco Carravetta, Roberto Maselli, Francesco Merlanti, Davide Fiore Bavaro, Antonio De Donno, Francesco Introna, Onofrio Caputi Iambrenghi

**Affiliations:** 1 Section of Legal Medicine, Department of Interdisciplinary Medicine, University of Bari, Piazza Giulio Cesare, 11, 70124 Bari, Italy; f.carravetta1@studenti.uniba.it (F.C.); r.maselli4@studenti.uniba.it (R.M.); francesco.merlanti@uniba.it (F.M.); antonio.dedonno@uniba.it (A.D.D.); francesco.introna@uniba.it (F.I.); 2 Section of General Surgery, Department of Emergency and Organ Transplantation, University of Bari, Piazza Giulio Cesare, 11, 70124 Bari, Italy; elpiniki.lagouvardou@policlinico.ba.it (E.L.); onofrio.caputiiambrenghi@uniba.it (O.C.I.); 3 Section of Pathology, Department of Emergency and Organ Transplantation (DETO), University of Bari, 70124 Bari, Italy; gerardo.cazzato@uniba.it; 4 Section of Infectious Diseases, Department of Biomedical Sciences and Human Oncology, University of Bari, 70124 Bari, Italy; davidebavaro@gmail.com

**Keywords:** necrotizing fasciitis, soft tissue infections, gastrointestinal endoscopy, autopsy, forensic pathology, malpractice, medical liability

## Abstract

Necrotizing fasciitis (NF) is an infection characterized by necrosis of the superficial muscle fascia and surrounding soft tissues. It usually occurs following skin breaches from penetrating traumas or high-degree burns. Less frequently, it could be related to major abdominal surgery. However, no cases of thigh NF after minor abdominal procedures have ever been reported. A previously healthy 59-year-old male patient underwent a colonoscopic polypectomy. After the procedure, the patient developed an increasing right groin pain. The CT scan showed a gas collection in the right retroperitoneum space and in the right thigh soft tissues. Thus, a right colon perforation was hypothesized, and the patient was moved to the nearest surgery department and underwent a right hemicolectomy procedure. During surgery, the right thigh was also incised and drained, with gas and pus leakage. Nevertheless, the right lower limb continued to swell, and signs of systemic infection appeared. Afterward, clinical conditions continued to worsen despite the drainage of the thigh and antibiotic therapy, and the patient died of septic shock after just two days. This case shows that, although rare, lower limb NF should be considered among the causes of early post-operative local painful symptoms.

## 1. Introduction

Necrotizing fasciitis (NF) is a rare yet potentially life-threatening soft-tissue infection, occurring with a prevalence of 0.3–15 cases every 100,000 and an annual incidence of 500–1000 cases [1,2]. NF is characterized by widespread necrosis of skin, subcutaneous fat, muscle fascia, and surrounding soft tissues which often evolves towards a systemic involvement followed by rapidly progressive septic shock.

The condition may affect any region of the body but frequently involves the abdominal wall, the perineum area, and limbs, especially the lower ones. Clinical presentation usually includes systemic symptoms such as fever, shivering, nausea, and diarrhea. Moreover, also local signs have been reported such as tenderness of the affected area, changes in skin color, stabbing pain, confined severe swelling, and palpable subcutaneous crepitus [3]. Patients with NF are frequently of advanced age and affected by existing comorbidities, such as a compromised immune system, or chronic illnesses such as diabetes mellitus (40–60%), chronic kidney disease, and alcohol/drug addiction [4,5]. Usually, the infection reaches the subcutaneous and muscular tissue through skin breaches such as penetrating traumas or high-degree burns. Nevertheless, there are few reported cases of NF without a cutaneous lesion, usually related to major abdominal surgery [6]. In the literature, there are only a few cases of NF following endoscopic procedures [7,8]. Due to its rapid progression, the gold standard treatment should be an aggressive surgical debridement of infected tissues, supported by an intravenous broad-spectrum antibiotic therapy. However, despite the early treatment, mortality remains very high, ranging from 29 to 100% [9,10].

## 2. Case Report

### 2.1. The Clinical, Surgical, and Radiological Data

A 59-year-old man without any known pre-existing health issues underwent a complete colonoscopy that pointed out the presence of three colic neoformations in the ascendant and transverse colon. Given this evidence, an endoscopic polypectomy was performed. The larger polyp was removed through diathermic loop resection and hemostatic clip placement, while the smaller one with a cold clamp. According to the operating report, the procedure was well-performed with clips properly placed, and no signs of intraoperative complications.

Soon after the patient returned home, he began to feel a stabbing pain in his right thigh. For this reason, after a few hours, he went to the emergency room, where an abdominal X-ray was initially performed pointing out the presence of free air contiguous at the sidewall of the ascending colon and at the right inguinal region. Given the radiological signs, a right colon perforation in the site of the previous polypectomy was hypothesized.

A CT scan was then necessary for deeper investigation. The radiological assessment showed mild thickening of the perivisceral adipose tissue and of the nearby lateroconal fascia. Moreover, a small amount of gas was identified near the iliopsoas and the inguinal canal. According to the radiologists, the bowel perforation was plugged by the right iliopsoas muscle.

The patient was then moved to another hospital where major abdominal surgery could be performed. The surgeons ordered another pre-operative CT scan to evaluate the evolution of the perforation. 

**Figure 1:**
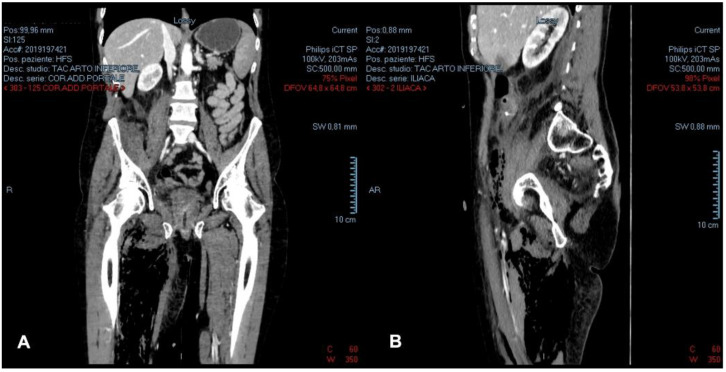
Pre-operative contrast-enhanced portal phase CT-scan of the abdomen and upper thighs: (**A**) coronal plane; (**B**) sagittal plane.

**Figure 2:**
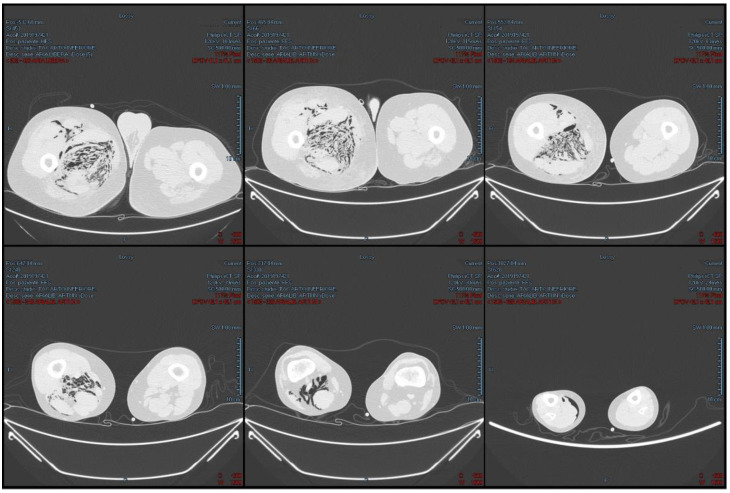
Pre-operative axial CT-scan of the lower limbs and lung window.

This second CT investigation showed that the inhomogeneous gathering detected inside the right iliopsoas muscle had spread to the lumbar and paraspinal muscle and the right thigh. For this reason, the analysis was extended to the right lower limb and showed an inhomogeneous gathering surrounding the right iliac vascular-nerve bundle; the gases located into the right iliopsoas muscles also reached the subcutaneous and muscular tissue of the right ankle. Imbibition of the soft tissues of the right thigh, with a fluid layer in the quadriceps, was also evident (Figure 1 and Figure 2).

The patient was then urgently transferred to the operating room. The surgeon performed a Hartmann resection of the right colon, a surgical toilette of the paracolic gutter, and a retrovescical pouch drainage. Furthermore, an incision with drainage of the right thigh was carried out with the leakage of pus and malodorous gas. 

In the following hours, a mild clinical improvement was observed, but immediately after, the right thigh started to swell, palpable crepitus was detected, and signs of systemic inflammation started to show up. The systemic impairment was confirmed by laboratory findings, that showed increases in CRP (17.26 mg/L) and PCT levels (1.13 ng/mL). Moreover, the blood culture was positive for Bacteroides fragilis (anaerobic bacteria).

Despite intravenous broad-spectrum antibiotic therapy and hemodialysis, the patient died just forty-two hours after the first endoscopic procedure.

### 2.2. Autopsy and Histopathology Findings

Three months later, the patient’s family requested a judicial investigation on the suspicion that the death was due to medical liability. Thus, a complete autopsy was ordered by the judicial authority and performed by cadaveric exhumation. 

Despite the time since death, decomposition phenomena were mild, probably because of the cold temperature, the burying in a zinc-lined chest, and the attack by hydrovorous molds. 

**Figure 3:**
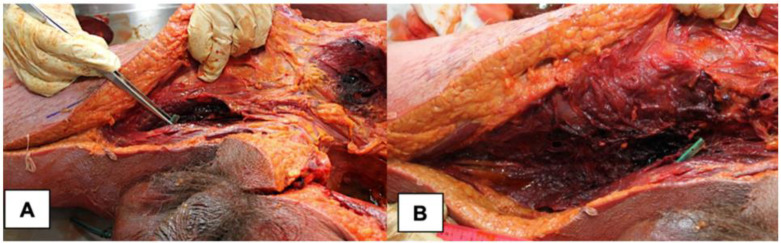
Post-mortem macroscopic appearance of NF. (**A**) Anatomical dissection of right iliac fossa and thigh. (**B**) Detail of right thigh muscle hemorrhagic infiltration.

The abdominal area showed no signs of putrefaction or infection at all, except for a hemorrhagic infiltration of the iliac fossa muscles. On the other hand, by cutting the right leg, a hemorrhagic infiltration of the thigh muscles and soft tissues was detected, and several muscle samples were taken for histological investigations (Figure 3).

**Figure 4:**
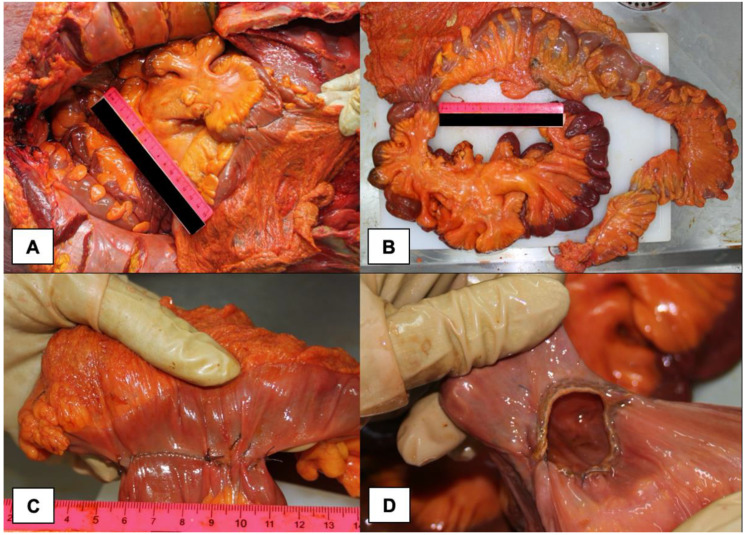
Macroscopic aspect of Hartmann resection of the right colon: (**A**) in situ evaluation of the colon; (**B**) ex situ evaluation of the colon; (**C**) detail of the suture; (**D**) endoluminal appearance of the suture.

The colic resection was flawless and showed no sign of dehiscence (Figure 4), indirectly confirming that the surgical procedure was correctly performed and the subject did not develop peritonitis when he was alive.

**Figure 5:**
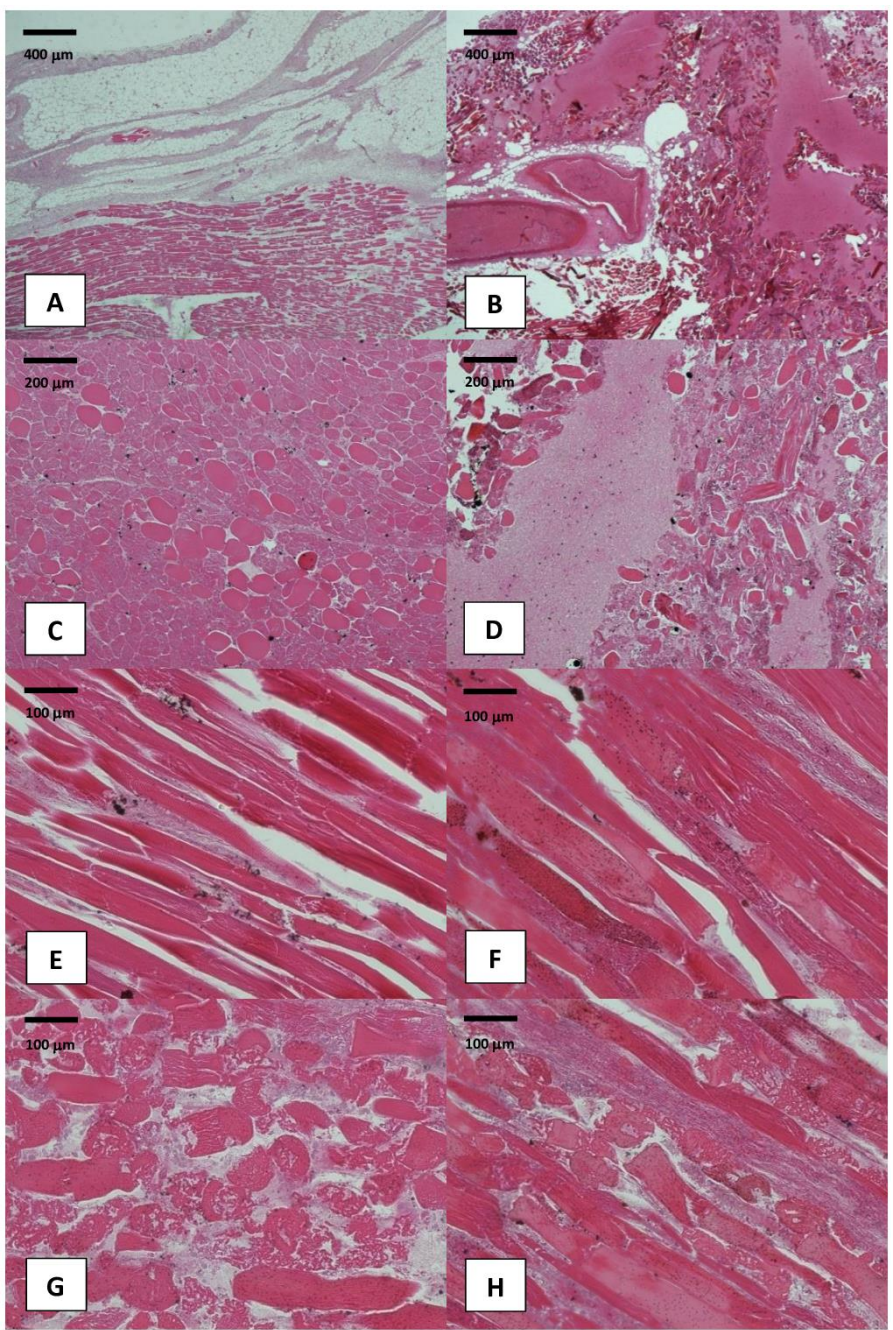
Histological images of thigh skeletal muscle samples (Hematoxylin and Eosin staining): (**A**–**D**) evidence of tissue emphysema, hemorrhagic infiltration, and fibrinoid tissue necrosis involving the thigh skeletal muscles (original magnification: 10× and 20×); (**E**–**H**) higher-power examination demonstrates bacterial colonization, edema, and myofibers’ disruption (original magnification: 40×).

According to histological criteria proposed by Stamenkovic and Lew for early recognition of NF [11], the microscopic observation of thigh soft tissue samples revealed the presence of hemorrhagic and inflammatory foci, with widespread signs of necrosis, tissue emphysema, fibrinous thrombi of arteries and veins coursing through the fascia, angiitis with fibrinoid necrosis of arterial and venous walls, and microorganisms colonization (Figure 5).

The diagnosis of septic shock secondary to necrotizing fasciitis was then confirmed.

## 3. Discussion and Conclusions

NF is an infective process sustained by polymicrobial flora of both aerobic and anaerobic bacteria (type I) by a single Gram-positive microorganism such as Streptococcus and methicillin-resistant Staphylococcus Aureus (type II), by monomicrobial infections involving Gram-negative bacteria, or by the Clostridium species (type III) [12,13,14,15].

The infection most commonly enters through skin lesions after penetrating trauma in an immunodeficiency scenario. However, in our case, radiological and autoptic evidence pointed out that NF of the right thigh was secondary to an iatrogenic colic perforation in an otherwise-healthy subject. The source of the infection was, therefore, in the abdominal cavity, but its local manifestations were exclusively in the right thigh. Indeed, the patient did not present any form of penetrating trauma, so the most likely hypothesis is that there was a bowel perforation during the colonoscopy. The air, blown into the intestine during the procedure, spread through the iatrogenic intestinal perforation into the retroperitoneum, and then down through the inguinal canal and into the thigh, transporting the intestinal microbial flora and causing necrotizing fasciitis. This unusual air migration (which was not in an anti-gravitational direction) may be due to the virtual nature of retroperitoneal space [16].

The correlation between pathological processes located in the abdominal or pelvic cavities and NF of lower limbs has been already described by different authors. 

Heamers et al. reported an unusual case of NF of the right thigh as a first presentation highly suggestive for rectal cancer; indeed, no other cases of secondary NF have been related to other kinds of colon carcinoma [16,17,18]. Nowicki et al. [19] reported a case of a sub-clinical necrotizing lower limb infection secondary to pelvic anastomotic leak and chronic corticosteroid use. Additionally, a case of NF of the lower limb secondary to colon perforation due to ingestion of foreign body (toothpick) is reported by Rupp et al. [20].

In the setting of surgical procedures, only one case report of NF after laparoscopy has been published [21].

However, to the best of our knowledge, NF secondary to an endoscopic procedure is a rare entity, and only a few reports are available in the scientific literature.

Sun et al. [8] illustrated a case of Fournier’s gangrene secondary to necrotizing pancreatitis after endoscopic retrograde cholangiopancreatography (ERCP), regarding a 66-year-old man with hypertension and coronary artery disease. Infectious symptoms appeared after two weeks from the ERCP. The patient underwent a surgical debridement for retroperitoneal abscess and emergent fasciotomy for necrotizing fasciitis of the right scrotum, with an uneventful postoperative course and a good recovery.

Lonie et al. [7] reported a case *Aeromonas sobria* NF of a lower limb following colonoscopy in the setting of ulcerative colitis. In that case, the 87-year-old female patient developed leg pain one week following the colonoscopy. Although she was old and a previously mesalazine-maintained patient, she survived thanks to empiric antibiotics therapy and an urgent surgical debridement of the leg infected tissues. 

Our patient, who was previously healthy and immunocompetent, is the first reported to die for an NF following an endoscopic procedure. In this case, lower limb pain appeared soon after the colonoscopy, and the patient died just forty-two hours later. Thus, this case confirms that the two goals in the management of NF are an early diagnosis by a specific examination of biochemical profile derangement, and culture/histology on affected tissue specimens [11,22,23,24,25], as well as a timely, aggressive therapy by surgical debridement [26,27]. Moreover, these diagnostic and therapeutic tools gain importance regardless of the patient’s immunocompetence, health, and age.

In the present case, the diagnosis was correctly formulated, but the treatment timing was not adequate, probably due to the rarity of this complication. This case report should alert clinicians about the potential infective complications of a post-polypectomy colic perforation. Indeed, NF may occur also distant from the surgical site and should be considered in the absence of alternative causes to explain early or spontaneous postoperative painful symptoms. 

Although the mortality of necrotizing fasciitis is very high and it is not certain that a timely and adequate treatment will save the patient’s life, this rare occurrence must be taken into consideration due to its serious clinical and medico-legal implications [28]. Indeed, NF misdiagnosis or mistreatment can promote medical malpractice litigation and lawsuits claiming compensation for patient damage. Several reasons may act as limitation of medical liability, such as patient-related (a medical history that could confer a risk of infection), healthcare-professionals-related (no-harm medical practice), and diseases-related factors (rapid progression and poor prognosis of NF) [29]. However, regardless of the outcome for the patient, timely recognition and management of NF seem to be some of the main factors for avoiding medical lawsuits and deciding malpractice in court [29].

## Data Availability

Not applicable.
